# HSP90AB1 as the Druggable Target of Maggot Extract Reverses Cisplatin Resistance in Ovarian Cancer

**DOI:** 10.1155/2023/9335440

**Published:** 2023-05-02

**Authors:** Daojuan Wang, Xun Tang, Jianguo Ruan, Zhengquan Zhu, Rong Wang, Yajing Weng, Yaling Zhang, Tingyu Wang, Ying Huang, Hongwei Wang, Zhenzi Su, Xiaoke Wu, Gaojian Tao, Yong Wang

**Affiliations:** ^1^The Affiliated Nanjing Drum Tower Hospital; State Key Laboratory of Analytical Chemistry for Life Science; and Jiangsu Key Laboratory of Molecular Medicine, Medical School of Nanjing University, Nanjing, Jiangsu 210008, China; ^2^Jiangsu Cancer Hospital & Jiangsu Institute of Cancer Research & The Affiliated Cancer Hospital of Nanjing Medical University, China; ^3^Department of Traditional Chinese Medicine, Nanjing Drum Tower Hospital, Nanjing University Medical School, Nanjing 210008, China; ^4^School of Medicine, Jiaxing University, Jiaxing 314001, China; ^5^Suzhou Cancer Center Core Laboratory, The Affiliated Suzhou Hospital of Nanjing Medical University, Suzhou, China; ^6^Department of Obstetrics and Gynecology, First Affiliated Hospital, Heilongjiang University of Chinese Medicine, Harbin 150040, China

## Abstract

Cisplatin resistance is a crucial factor affecting ovarian cancer patient's survival rate, but the primary mechanism underlying cisplatin resistance in ovarian cancer remains unclear, and this prevents the optimal use of cisplatin therapy. Maggot extract (ME) is used in traditional Chinese medicine for patients with comas and patients with gastric cancer when combined with other drug treatments. In this study, we investigated whether ME enhances the sensitivity of ovarian cancer cells to cisplatin. Two ovarian cancer cells—A2780/CDDP and SKOV3/CDDP—were treated with cisplatin and ME in vitro. SKOV3/CDDP cells that stably expressed luciferase were subcutaneously or intraperitoneally injected into BALB/c nude mice to establish a xenograft model, and this was followed by ME/cisplatin treatment. In the presence of cisplatin, ME treatment effectively suppressed the growth and metastasis of cisplatin-resistant ovarian cancer in vivo and in vitro. RNA-sequencing data showed that HSP90AB1 and IGF1R were markedly increased in A2780/CDDP cells. ME treatment markedly decreased the expression of HSP90AB1 and IGF1R, thereby increasing the expression of the proapoptotic proteins p-p53, BAX, and p-H2AX, while the opposite effects were observed for the antiapoptotic protein BCL2. Inhibition of HSP90 ATPase was more beneficial against ovarian cancer in the presence of ME treatment. In turn, HSP90AB1 overexpression effectively inhibited the effect of ME in promoting the increased expression of apoptotic proteins and DNA damage response proteins in SKOV3/CDDP cells. Inhibition of cisplatin-induced apoptosis and DNA damage by HSP90AB1 overexpression confers chemoresistance in ovarian cancer. ME can enhance the sensitivity of ovarian cancer cells to cisplatin toxicity by inhibiting HSP90AB1/IGF1R interactions, and this might represent a novel target for overcoming cisplatin resistance in ovarian cancer chemotherapy.

## 1. Introduction

Ovarian cancer is one of the most lethal cancers among gynecological malignancies and has high recurrence rates [1, 2]. Accumulating evidence has shown that the survival rate of ovarian cancer patients is low, partly due to late diagnosis at an advanced stage, a lack of curative front-line treatments, and drug resistance [3]. The standard treatment option for ovarian cancer is a combination of aggressive surgical ablation and platinum-based chemotherapy [4]. Cisplatin is a widely used platinum compound and is a first-line chemotherapeutic agent for ovarian cancer that causes tumor cell death mainly by inducing cellular DNA damage [5, 6]. However, intrinsic or acquired cisplatin resistance is an important factor causing the decreased efficacy of chemotherapy [7], and this is still a major challenge due to a poor understanding of the molecular mechanisms responsible for tumor metastasis and chemoresistance [8]. In recent years, numerous studies have shown that chemoresistance involves different mechanisms such as immune tolerance, mutations in drug targets, and abnormalities in DNA damage and repair systems [9–11]. In light of the issues related to cisplatin treatment, traditional Chinese medicine has become widely accepted as an alternative treatment for cancer [12, 13], and this study is aimed at determining whether traditional Chinese medicine can effectively enhance the sensitivity of ovarian cancer to cisplatin chemotherapy.

Maggots, known in traditional Chinese medicine as “Wu Gu Chong”, are the larvae of *Lucilia sericata*, a species of blowfly found in China [14]. Previous research on maggots indicates that their excretions/secretions can effectively suppress multiple inflammatory responses mediated by neutrophils, including chemotaxis, degranulation, respiratory bursts, and enhanced integrin expression [15]. Increasing evidence suggests that maggot therapy has advantages over conventional treatments, especially for the treatment of wounds infected by drug-resistant bacteria [16, 17]. In addition, maggot extract (ME) combined with other drugs has been shown to be beneficial for patients in a coma and in patients with gastric cancer [18]. However, the mechanisms by which ME inhibits tumors and induces apoptosis in cancer cells have not been fully elucidated.

Heat shock protein 90 (HSP90) is a highly conserved molecular chaperone that stabilizes and activates various proteins [19], and recent studies have shown that the ATPase activity of HSP90 is critical for the chaperone cycle of the HSP90 machinery [20]. HSP90 alpha and HSP90 beta are the two major HSP90 isoforms present in the cytosol, and they both support the correct folding of proteins, stabilize protein structures, and participate in cell signal transduction and transcription regulation [21, 22]. HSP90 alpha, class B, member 1 (HSP90AB1) is a crucial facilitator of oncogene activation and cancer cell survival because the HSP90AB1 chaperone machinery in cancer cells can protect large amounts of mutated and overexpressed oncogenic proteins from misfolding and degradation [23]. However, inhibition of HSP90AB1 in cancer cells leads to extensive degradation of oncogenic proteins [24, 25]. Increased expression of HSP90AB1 is strongly associated with tumor metastasis due to its ability to sustain the activity of an integrin-linked kinase, chaperone focal adhesion kinase, and the receptor tyrosine kinases ErbB2 and C-met (a hepatocyte growth factor receptor) [26, 27]. In addition, HSP90AB1 overexpression plays an integral role in the increased tolerance of gastric cancer cells to chemotherapeutic agents, which negatively affects the prognosis of patients [24].

Insulin-like growth factors (IGFs) are peptide hormones that are implicated in the development of several types of cancers [28, 29]. The IGF signaling system comprises four components—insulin, IGF1, IGF2, and insulin-like growth factor binding proteins (IGFBPs). Three cellular membrane-spanning receptors—insulin receptor, IFG1 receptor (IGF1R), and IGF2 receptor (IGF2R) [30]—medicate IGF signaling. The expression and activity of IGF1R are increased in numerous tumor types, including ovarian cancer and rhabdomyosarcoma [31]. Recent studies have shown that IGF1R is associated with the development of a variety of human malignancies such as colorectal cancer and pancreatic adenocarcinoma [32, 33], and molecular and clinical evidence suggests that IGF1R plays a critical role in the proliferation, migration, angiogenesis, and apoptosis of cancer cells [34]. Previous studies have shown that nuclear IGF1R is associated with other recombinational DNA repair pathways, and IGF1R inhibition can decrease homologous repair and delay the resolution of *γ*H2AX foci [35]. Moreover, the expression of nuclear IGF1R in tumors has been shown to be involved in chemotherapy and radiotherapy resistance [36]. In addition, a recent study has reported that the sulfur-containing nucleophiles glutathione (GSH) and nuclear metallothionein can bind cisplatin and inactivate the drug, thereby increasing drug resistance [37, 38].

Recent studies have shown that angiogenesis and cancer metastasis are associated with increased HSP90AB1 and IGF1R expression in numerous solid tumors [24, 39, 40], and in this study, we explored the potential interaction between HSP90AB1 and IGF1R in ovarian cancer tissue and ovarian cancer cell lines that might mediate cisplatin resistance. Furthermore, we examined whether ME could regulate the HSP90AB1/IGF1R interaction so as to overcome cisplatin resistance and induce apoptosis in ovarian cancer cells. Our study might therefore provide new insights into therapeutic strategies for ovarian cancer.

## 2. Methods

### 2.1. ME Preparation

Maggot powder was obtained from the Jiangsu Key Laboratory of Molecular Medicine, Nanjing University. The ME homogenate was prepared by adding four volumes of PBS and then centrifuging at 15,000 × g for 10 min. The supernatant was collected and incubated in a water bath at 70°C for 30 s followed by centrifugation again at 15,000 × g for 10 min. The supernatant was collected and passed through a 0.22 *μ*m filter (Millipore, USA) [14].

### 2.2. Ovarian Cancer Cell Culture

The human ovarian cancer cell line A2780 (RRID: CVCL_0134) was purchased from Shanghai Pituo Biological Technology Co., Ltd. (China), and the cell line SKOV3 (RRID: CVCL_0532) was purchased from Solarbio Science & Technology Co., Ltd. (China). The cisplatin-resistant ovarian cancer cell lines A2780/CDDP (RRID: CVCL_D619) and SKOV3/CDDP (RRID: CVCL_D622) were purchased from BeNa Culture Collection (BNCC) (China). A2780 and A2780/CDDP were cultured in RPMI-1640 medium (Gibco, USA) containing 10% FBS (Gibco), 100 U/mL of penicillin, and 100 U/mL of streptomycin (Gibco). SKOV3 and SKOV3/CDDP were cultured in DMEM medium (Gibco) containing 10% FBS (Gibco), 100 U/mL of penicillin, and 100 U/mL of streptomycin (Gibco). Cisplatin (1 *μ*g/mL) was added to the medium of the cisplatin-resistant cell lines to maintain resistance. All experiments were performed with mycoplasma-free cells.

### 2.3. RNA-Seq Data Analysis

The total RNA from A2780 and A2780/CDDP cells was extracted using an RNA Miniprep kit (Beyotime, China) according to the manufacturer's instructions. Next-generation sequencing analysis was performed on the BGISEQ-500 platform by BGI Genomic Services. The raw data contained low-quality reads, a large number of unknown bases, and the sequence of the adaptor. To decrease data noise, we further filtered these reads prior to downstream analysis. First, the RNA-seq library quality was assessed using the Fast Quality Control v0.11.5 software. Then the reads were subjected to standard quality control criteria. The filtered reads were considered “clean reads” and were stored in FASTQ format. Standard bioinformatics analysis included differential gene expression, volcano plots, heat maps, and Gene Ontology (GO) and KEGG pathway analyses, all of which were performed by BGI Genomic Services. The differentially expressed cancer target protein interaction network was established using the STRING database. All of the data has been uploaded to the GEO database (GSE206649).

### 2.4. Luciferase Reporter Assay

Cells were seeded in 6-well plates and infected with lentiviral particles to express luciferase (GeneChem, Shanghai, China), and HitransG P was added to the growth medium after the cell confluence reached 50%. After 72 h, the cells expressing luciferase were amplified and then seeded in 96-well plates. Finally, cells were treated with 4 *μ*g/ml puromycin to screen for cells that express luciferase.

### 2.5. Lentiviral Vector Preparation and Infection

HSP90AB1 lentiviral Ubi-MCS-3FLAG-SV40-EGFP-IRES-puromycin for overexpression in human cells was purchased from GeneChem (Shanghai, China). SKOV3/CDDP cells were seeded in 6-well plates, and when the cell confluence reached 50%, the cells were infected with lentiviral particles for 72 h.

### 2.6. Cell Viability Assay

All of the cell lines were seeded in 96-well plates (1 × 10^4^ cells/well). The next day, the cells were treated with various concentrations of cisplatin for 72 h or with ME for 48 h. Cell viability was measured using a Cell Counting Kit-8 (CCK8, A311-02-AA, Vazyme Biotech, China). After incubating with the reagent for 2 h, the absorbance at 450 nm was determined using a microplate reader.

For the cells expressing luciferase, we treated the cells with 3.2 *μ*g/ml cisplatin or different concentrations of ME for 48 h. Cell viability was measured on a GloMax® 96 Microplate Luminometer (E6521, USA) using Living Image software (LB 983 NC100, Germany).

### 2.7. Wound-Healing Assay

A2780/CDDP and SKOV3/CDDP cells (1 × 10^5^ cells/well) were seeded into 12-well plates. At 80% confluence, the cells were scraped with a 10 *μ*l sterile pipette tip and then washed with PBS. The scratched cells were further cultured with cisplatin, ME, or both cisplatin and ME for another 48 h. Images were captured at the end of 48 h under an inverted microscope (Mshot, China), and the wound closure rate was measured and analyzed with Image J.

### 2.8. Transwell Invasion Assay

The cellular invasion assay was performed using 24-well transwell plates (8.0 *μ*m pore size; Merck Millipore, USA). A2780 and A2780/CDDP cells were seeded into the upper chamber of 24-well transwell plates in 200 *μ*L RPMI-1640 medium containing 2% FBS, and the bottom chamber was filled with 500 *μ*L of complete RPMI-1640 medium containing 10% FBS. Cells were divided into four groups—blank, cisplatin, ME, and cisplatin+ME. The cisplatin and ME were added to the upper chamber, and the cells were allowed to migrate for 48 h. The cells were then washed, fixed with methyl alcohol, and stained with 10% Giemsa stain (Solarbio, China) for 10 min. The invading cells on the bottom of the membrane were photographed using an inverted microscope (Mshot, China). All of the experiments were performed in triplicate.

### 2.9. Annexin V-FITC/PI Assay

A2780/CDDP were seeded in 24-well plates at a density of 1 × 10^5^ cells/well and cultured in full culture medium for 24 h. Next, cells were treated with cisplatin, ME, or both for 72 h. A dual annexin V-fluorescein isothiocyanate/propidium iodide (FITC/PI) binding assay was used to detect apoptotic cells (C1062L, Beyotime, China), and the cells were photographed using an Olympus laser scanning confocal microscope (FV3000).

### 2.10. Flow Cytometry Analysis

A2780/CDDP and SKOV3/CDDP cells were seeded in 6-well plates at a density of 3 × 10^5^ cells/well for 24 h and then treated with 3.2 *μ*g/ml cisplatin or with 6 ml/mg or 8 mg/ml of ME or both for 72 h. All of the cells were collected in a flow tube and stained with dual annexin V-FITC/PI. The ratio of apoptotic cells in the different groups was analyzed using a flow cytometry instrument (FACS Calibur, BD, USA).

### 2.11. Glutathione (GSH) Assay

All of the cells were seeded in 12-well plates and treated with 3.2 *μ*g/ml cisplatin, 6 mg/ml ME, or both for 48 h. Cells were collected and washed with PBS two times then sonicated at 200 W for 3 s with 10 s intervals for a total of 30 cycles. After centrifugation, the supernatant was collected and stored at 4°C. The cell GSH content was detected with a GSH analysis kit according to the manufacturer's instructions (Solarbio Life Sciences, China).

### 2.12. In Vivo Xenograft Model

Six-week-old female BALB/c nude mice (*n* = 32) weighing 16–18 g were obtained from the Nanjing Junke Biotechnology Corporation. The mice were housed in a temperature- and humidity-controlled specific pathogen-free environment (Jiangsu Key Laboratory of Molecular Medicine) with a 12 h light/dark cycle.

Half of the mice were subcutaneously injected with 1 × 10^7^ SKOV3/CDDP cells (100 *μ*L of saline) into the flank, while the other half of the mice were intraperitoneally injected with 1 × 10^7^ SKOV3/CDDP cells (100 *μ*L of saline). Seven days after injection, the mice were anesthetized and intraperitoneally injected with D-luciferin (150 mg/kg body weight), and then 10 min later imaging data were acquired and analyzed with Living Image software (LB 983 NC100, Germany). The mice were then randomly divided into four groups (con, cis, ME, cis+ME, *n* = 4 for each group). The mice in the cisplatin group were intraperitoneally injected with 5 mg/kg cisplatin once a week for 4 weeks; the mice in the ME group were given 1 g/kg ME orally three times a week for 4 weeks; and the mice in the cis+ME group received both. After 5 weeks of cell inoculation, the mice were anesthetized and injected intraperitoneally with D-luciferin (150 mg/kg body weight), and 10 min later, imaging data were acquired and analyzed by Living Image software (LB 983 NC100, Germany). Then the mice were euthanized to assess the tumor load, and tumors were collected for molecular analysis.

### 2.13. Immunofluorescence

A2780/CDDP cells were seeded onto 12-well plates with round glass. Following treatment with cisplatin, ME, or both, the cells were fixed, permeabilized with 0.3% Triton X-100, and blocked with 3% BSA. The cells were then incubated with antibodies against p-p53 (Abcam, UK) overnight at 4°C. The cells were incubated with fluorescent secondary antibodies at room temperature for 1.5 h, and nuclei were counterstained with DAPI (Beyotime, China) for 30 min. Images were photographed using an Olympus laser scanning confocal microscope (FV3000).

### 2.14. Immunohistochemistry

Tumors were fixed, embedded in paraffin, and processed on slides for immunohistochemistry staining. Tumor sections were stained with specific antibodies against Ki67 (1 : 250 dilution, Abcam, UK) and CD31 (1 : 100 dilution, Abclonal, China). The sections were subsequently incubated with an HRP-conjugated secondary goat anti-rabbit IgG (H+L), and images were captured using an optical microscope (Leica Microsystems, Germany).

### 2.15. Western Blot

Cell lysates were separated by electrophoresis on 12% SDS-PAGE gels, and the proteins were transferred to polyvinylidene difluoride membranes (IPVH00010, Merck Millipore, USA) and then incubated with primary antibodies overnight at 4°C. Next, the membranes were probed with the appropriate secondary antibodies for 1.5 h. The blots were visualized using chemiluminescent detection (Vazyme Biotech, China) and analyzed by Image J software. Loading was normalized with GAPDH.

### 2.16. Quantitative Real-Time PCR (qRT-PCR)

Total RNA from tissues and cells was extracted using an RNA Miniprep kit (Beyotime, China) according to the instructions, and cDNA was synthesized with a reverse transcription kit (Vazyme Biotech, China). SYBR Green PCR Master Mix (Vazyme, China) was used to analyze the relative gene expression, and qRT-PCR was performed with the ABI Viia 7 Real-Time PCR system (ABI, USA). *β*-Actin was used as the internal control, and the primers are shown in Table 1. The critical threshold cycle (Ct) value was determined for each reaction, which was transformed into relative quantification data using the 2^-ΔΔCt^ method.

### 2.17. Coimmunoprecipitation

The SKOV3/CDDP cell lysates were first immunoprecipitated with antibodies against HSP90AB1, IGF1R, or isoform-matched immunoglobulin (IgG), and then the immunoprecipitants were assayed by western blotting with antibodies against IGF1R and HSP90AB1.

### 2.18. Statistics

All statistical analyses were performed with GraphPad Prism 7. Comparisons between two groups for statistical significance were assessed using a two-tailed Student's t-test. The differences between multiple groups were calculated using a one-way ANOVA followed by Tukey's post hoc test. A *p* value <0.05 was considered statistically significant, and all the data presented are from at least three independent experiments.

## 3. Results

### 3.1. ME Enhances the Sensitivity of Ovarian Cancer Cells with Chemoresistance to Cisplatin

**Figure 1:**
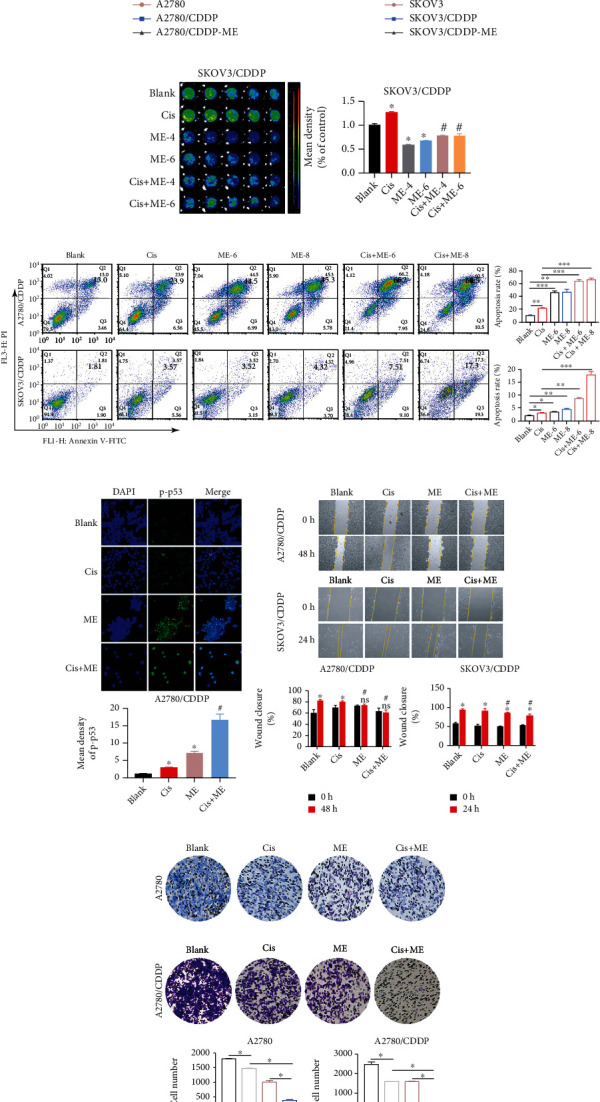
ME enhances the sensitivity of A2780/CDDP and SKOV3/CDDP cells to cisplatin (Cis). Cells were treated with different concentrations of cisplatin with and without ME (6 mg/ml) for 48 h. (a) The viability of A2780 and A2780/CDDP cells was assayed using CCK8 kits. (b) The viability of SKOV3 and SKOV3/CDDP cells was assayed. SKOV3/CDDP cells were infected with lentiviral particles to express luciferase, and then cells were treated with cisplatin (3.2 *μ*g/ml) and/or ME (4 mg/ml or 6 mg/ml) for 48 h followed by 150 *μ*g/ml D-luciferin for 10 min. (c) The luciferase-positive SKOV3/CDDP cells were analyzed using Living Image software and a GloMax® 96 Microplate Luminometer. The quantification of luciferase-positive cells is shown on the right. ^∗^*p* ≤ 0.05 vs. blank; ^#^*p* ≤ 0.05 vs. cisplatin; (d) A2780/CDDP and SKOV3/CDDP cells were double stained with Annexin V-FITC and PI then analyzed using flow cytometry, and the quantitative analysis was located in the right. ^∗^*p* ≤ 0.05, ^∗∗^*p* ≤ 0.01, ^∗∗∗^*p* ≤ 0.001. (e) Levels of p-p53 in A2780/DDP cells were measured by immunofluorescence staining (400x magnification). The panel under the staining shows the quantitative analysis of p-p53 in A2780/CDDP cells. The nuclei are stained with DAPI. ^∗^*p* ≤ 0.05 vs. blank; ^#^*p* ≤ 0.05 vs. cisplatin. (f) Representative images of the wound healing assays in A2780/CDDP and SKOV3/CDDP cells are shown, and the panels under the images show the wound closure rate. ^∗^*p* ≤ 0.05 vs. 0 h; ^#^*p* ≤ 0.05 vs. blank; ns means no significance vs. 0 h. (g) The transwell assay was used to analyze the invasion of A2780 and A2780/CDDP cells. The invasive cell number was quantified using ImageJ and located under the images. ^∗^*p* ≤ 0.05. Three independent experiments were performed with similar results. Data are shown as the mean ± SEM.

A2780, A2780/CDDP, SKOV3, and SKOV3/CDDP cells were treated with different concentrations of cisplatin to verify the drug resistance of the ovarian cancer cells. The results showed that cisplatin concentrations corresponding to 50% cell viability in A2780 and SKOV3 cells were 4.0 *μ*g/ml and 3.1 *μ*g/ml, respectively. As expected, cisplatin concentrations corresponding to 50% cell viability of A2780/CDDP and SKOV3/CDDP cells were 7.8 *μ*g/ml and 16 *μ*g/ml, respectively (Figures 1(a) and 1(b)). Furthermore, we treated A2780/CDPP and SKOV3/CDDP cells with ME to investigate whether ME sensitizes ovarian cancer cells to cisplatin. The cisplatin concentrations corresponding to 50% cell viability of A2780/CDPP and SKOV3/CDDP cells were 2.0 *μ*g/ml and 6.5 *μ*g/ml, respectively, when combined with ME treatment (Figures 1(a) and 1(b)). Our findings demonstrate that ME treatment can significantly enhance the sensitivity of chemoresistant ovarian cancer cells to cisplatin.

### 3.2. ME Combined with Cisplatin Therapy Promotes Apoptosis and Suppresses Migration in Chemoresistant Ovarian Cancer Cells

Tumor chemoresistance is a major issue for ovarian cancer therapy. To further investigate whether ME treatment can overcome ovarian cancer chemoresistance, we treated ovarian cancer cells with ME and/or cisplatin. SKOV3/CDDP cells were transfected with a lentivirus expressing luciferase and analyzed using Living Image software and a GloMax® 96 Microplate Luminometer. We first screened for luciferase-expressing cells using puromycin (Supplementary Figure 1). The results showed that the cell viability was markedly decreased in the combined cisplatin and ME group compared to cisplatin treatment alone in both cisplatin-resistant and non-resistant cells (Figure 1(c)). We next analyzed cell apoptosis rates using an Annexin V/PI assay. Flow cytometry analysis of A2780/CDDP and SKOV3/CDDP cells with cisplatin treatment showed a two-fold increase in apoptosis compared to the blank group. As expected, the cell apoptosis rates of A2780/CDDP and SKOV3/DDP with both ME and cisplatin treatments were five-fold and ten-fold greater, respectively, than the blank groups (Figure 1(d)). The results of fluorescence confocal microscopy were consistent with the flow cytometry results (Supplementary Figure 2).

To further confirm that ME can enhance the cisplatin sensitivity of chemoresistant ovarian cancer cells, the expression of p-p53 in A2780/CDDP cells was analyzed using immunofluorescent staining and was shown to be significantly increased with the combination of ME and cisplatin and was higher than ME or cisplatin treatment alone (Figure 1(e)). The wound healing assay showed that cisplatin had little effect on the migration of A2780/CDDP and SKOV3/CDDP cells (Figure 1(f)). However, ME treatment alone or combined with cisplatin significantly suppressed the migration of A2780/CDDP and SKOV3/CDDP cells. The inhibitory effect of ME treatment combined with cisplatin on cell migration was more obvious (Figure 1(f)). The transwell assay showed that ME combined with cisplatin effectively inhibited the invasion of A2780 cells. Moreover, ME combined with cisplatin treatment significantly inhibited the invasion of A2780/CDDP cells more effectively than cisplatin or ME treatment alone (Figure 1(g)). Taken together, these results indicate that ME combined with cisplatin can overcome chemoresistance, induce apoptosis, and inhibit migration in ovarian cancer cells.

### 3.3. Me Treatment Inhibits the Growth of Cisplatin-Resistant Ovarian Tumors In Vivo

**Figure 2:**
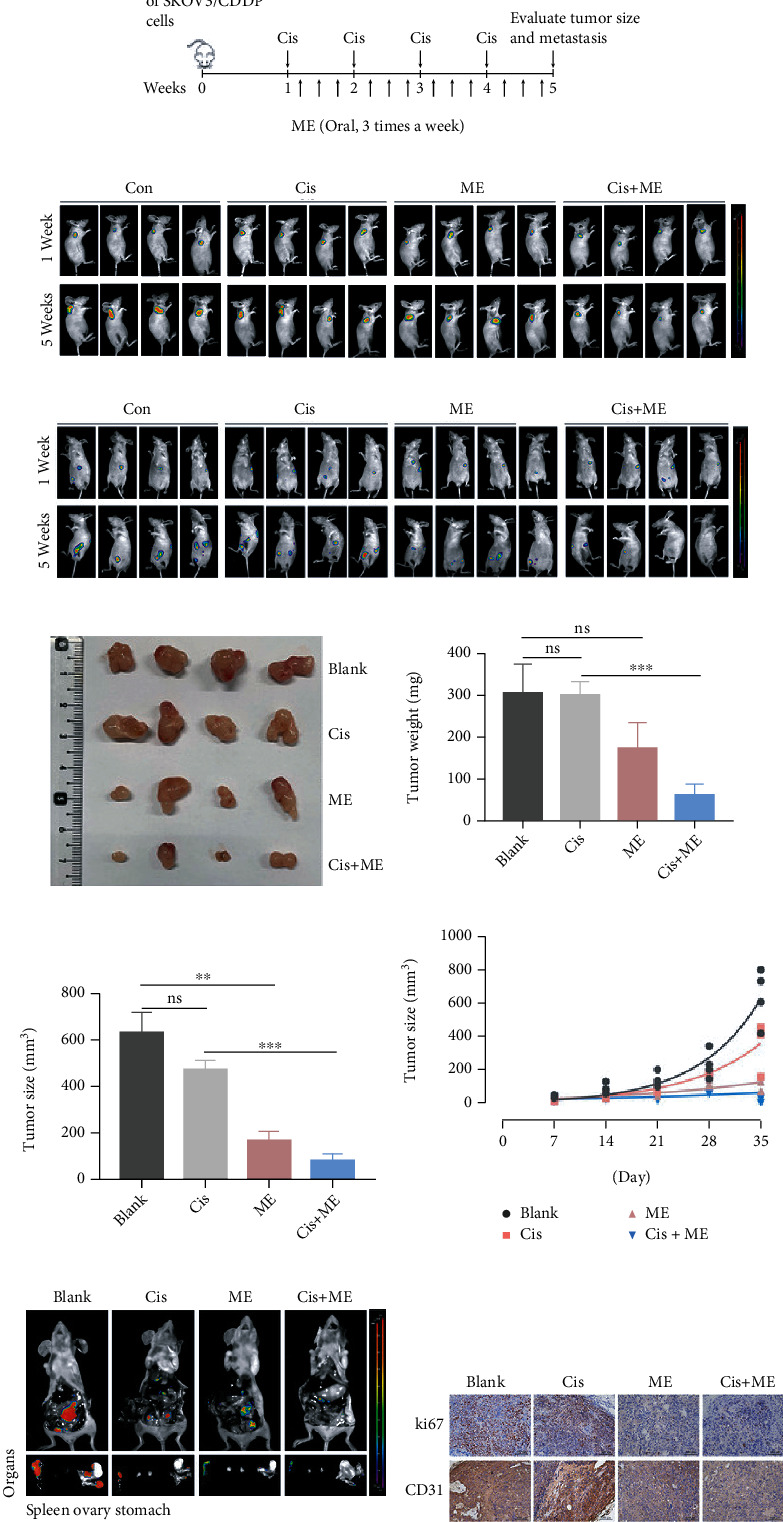
ME combined with cisplatin effectively inhibits cisplatin-resistant ovarian tumor growth. (a) Nude mice were implanted with SKOV3/CDDP cells by subcutaneous injection or intraperitoneal injection. The two kinds of tumor-bearing mice were divided into four groups (*n* = 4 mice/group). The animals were treated with intraperitoneal injection of cisplatin (5 mg/kg) every seven days for a total of four times. Mice receiving ME treatments were administered with ME (1 g/kg, three times a week, orally) for four consecutive weeks. After 1 and 5 weeks of cell inoculation, mice were anesthetized and live images were acquired. (b) Mice with subcutaneous tumors were injected intraperitoneally with D-luciferin (150 mg/kg body weight), and 10 min later imaging data were acquired and analyzed using Living Image software. (c) Intraperitoneal tumor-bearing mice were injected intraperitoneally with D-luciferin and analyzed using Living Image software. (d) Photographs of the morphology of the tumors from each treatment group are shown. (e) The mean tumor weight in the different groups. (f) The mean tumor size in the different groups. (g) On day 7, 14, 21, 28, and 35 of the subcutaneous tumors, tumor size was measured, and the tumor growth curve was shown. (h) Intraperitoneal tumor-bearing mice were dissected. Imaging data of the spleen, bilateral ovaries, and stomach were acquired and analyzed using Living Image software. (i) The expression of Ki67 and CD31 in each group of tumors was analyzed using immunohistochemical staining. Three independent experiments were performed with similar results. Data are shown as the mean ± SEM. ^∗∗^*p* ≤ 0.01, ^∗∗∗^*p* ≤ 0.001 vs. blank; ns means no significance.

Because we observed that ME had a striking inhibitory effect on cisplatin-resistant cells, we further established a xenograft model by subcutaneous or intraperitoneal injection of SKOV3/CDDP cells in BALB/c nude mice and then treated the mice with ME, cisplatin, or both (Figure 2(a)). SKOV3/CDDP cells that stably expressed luciferase were used to detect tumors in mice by bioluminescence imaging. One week after cell inoculation, tumors were analyzed by bioluminescence imaging, and then the treatment groups were intraperitoneally injected with cisplatin, intragastrically administrated with ME, or given both treatments (Figures 2(b) and (c)). By 5 weeks after treatment, the luciferase-positive areas were clearly decreased after treatment with ME combined with cisplatin, but not with cisplatin or ME treatment alone (Figures 2(b) and 2(c)). In addition, the sizes and weights of tumors from the subcutaneous xenograft model were significantly decreased with ME combined with cisplatin (Figures 2(d)–2(f) and Supplementary Figure 3A), and the tumor growth rate in the ME combined with cisplatin treatment group was significantly lower than other groups (Figure 2(g)). The intraperitoneally injected tumor cells predominantly colonized the intestine, spleen, and stomach (Figure 2(h) and Supplementary Figure 3B). We next analyzed Ki67 and CD31 expression in cisplatin-resistant tumors using immunohistochemical staining, and these were markedly downregulated in the ME alone and ME combined with cisplatin treatment groups (Figure 2(i)). These findings indicate that ME treatment can enhance the sensitivity of ovarian cancer to cisplatin and can inhibit tumor growth and metastasis.

### 3.4. Correlation and Enrichment Analyses of HSP90AB1 and IGF1R in Ovarian Cancer Cells by RNA Sequencing

**Figure 3:**
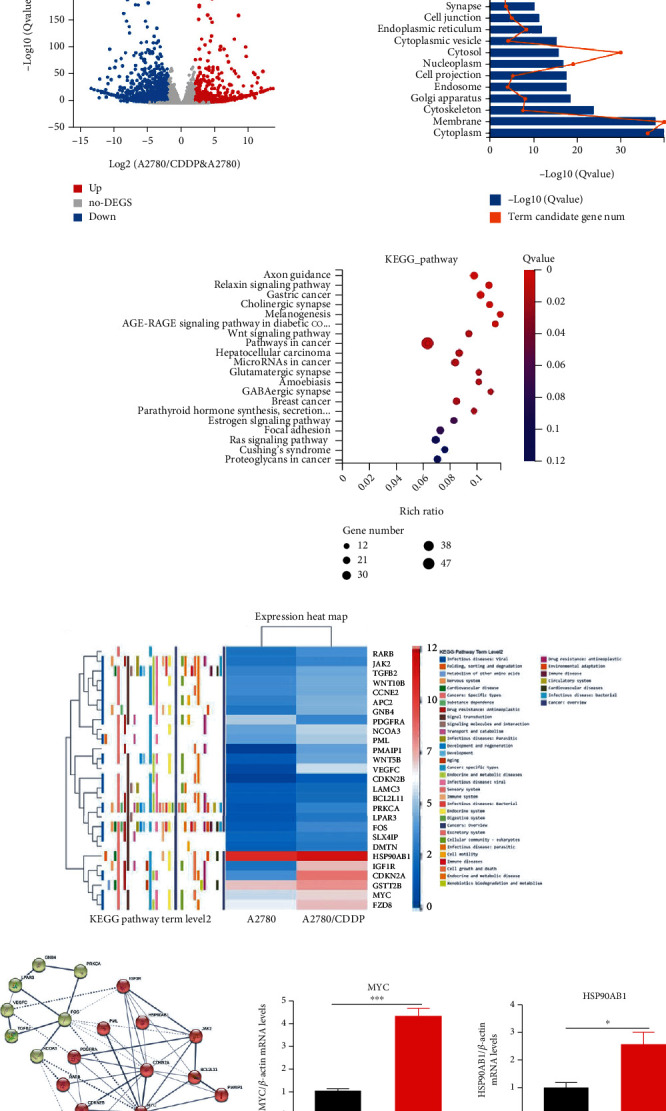
Enrichment analysis of DEGs in A2780/CDDP cells based on high-throughput RNA sequencing. The total RNA from A2780 and A2780/DDP cells was extracted using RNA Miniprep kit reagents. Next-generation sequencing analysis was performed on the BGISEQ-500 platform by BGI Genomic Services. (a) Volcano plot showing significantly upregulated genes (red dots) and downregulated genes (blue dots). (b) A2780 vs. A2780/CDDP Gene Ontology (GO) analysis on a cellular component of DEGs. (c) A2780 vs. A2780/CDDP KEGG pathway enrichment analysis of DEGs. (d) The heat map shows the relative transcript levels of the DEGs in A2780 and A2780/CDDP cells. (e) The protein–protein interaction network shows the upregulated DEGs from A2780/CDDP cells compared with A2780 cells. (f) qRT-PCR analyses of the mRNA levels of *MYC* in A2780 cells and A2780/CDDP cells. (g) qRT-PCR analyses of the mRNA levels of *HSP90AB1* in A2780 cells and A2780/CDDP cells. Three independent experiments were performed with similar results. Data are shown as the mean ± SEM. ^∗^*p* ≤ 0.05, ^∗∗∗^*p* ≤ 0.001.

To reveal the molecular mechanism underlying the development of chemoresistance in ovarian carcinoma, a differentially expressed gene (DEG) analysis was performed to identify the difference in gene expression between A2780 and A2780/CDDP cells. By RNA sequencing (RNA-seq), a total of 45,573,892 raw reads were obtained from A2780 and A2780/CDDP cells. After cleaning and quality control, the number of clean reads for further analysis was reduced to 44,482,827 and 44,710.501 reads, respectively. A total of 6,166 DEGs (|fold change| >2 and *p* < 0.05) were detected between the A2780 and A2780/CDDP cDNA libraries, of which 3,037 genes were upregulated (higher expression in A2780/CDDP cells) and 3,129 genes were downregulated (lower expression in A2780/CDDP cells) (Figure 3(a)). This result indicated the great variation in gene expression between A2780 cells and the chemoresistant A2780/CDDP cells. We further explored the potential molecular functions based on the top 1,038 DEGs using the phyper function in R software to perform the enrichment analysis. We performed GO analysis on the cellular component of DEGs. We found DEGs were significantly enriched in cytoplasm, membrane, and cytoskeleton (Figure 3(b)). In addition, KEGG pathway analysis indicated the enrichment and crosstalk of 47 genes in cancer pathways (Figure 3(c)).

To identify novel upregulated genes related to cisplatin resistance, ten novel genes were identified that were significantly upregulated in A2780/CDDP cells compared with A2780 cells (Figure 3(d)). In particular, the expression of *IGF1R* and *CDKN2A* in A2780/CDDP cells was over 20-fold higher compared to that of A2780 cells, and the results of the qRT-PCR analysis were consistent with this finding (Figure 3(d) and Supplementary Figure 4). The IGF1R and CDKN2A proteins are associated with cell growth and death. Significantly, *HSP90AB1*, a prognostic marker of ovarian cancer, was highly expressed in both A2780 and A2780/CDDP cells (Figure 3(d)). To identify potential interaction partners of HSP90AB1, a human protein-protein interaction network analysis was performed using the STRING database (Figure 3(e)). The data indicated that HSP90AB1 likely interacts with IGF1R, which is also associated with cancer cell growth and death (Figure 3(e)). In addition, IGF1R can also potentially interact with MYC. Consistent with this, the mRNA levels of *MYC* and *HSP90AB1* as analyzed by QPCR were significantly increased (Figures 3(f) and 3(g)). These findings strongly support the hypothesis that ovarian cancer chemoresistance is associated with the overexpression of HSP90AB1 and IGF1R.

### 3.5. ME Treatment Attenuates the HSP90AB1/IGF1R Pathway and Inhibits DNA Damage Repair in Cisplatin-Resistant Cells

**Figure 4:**
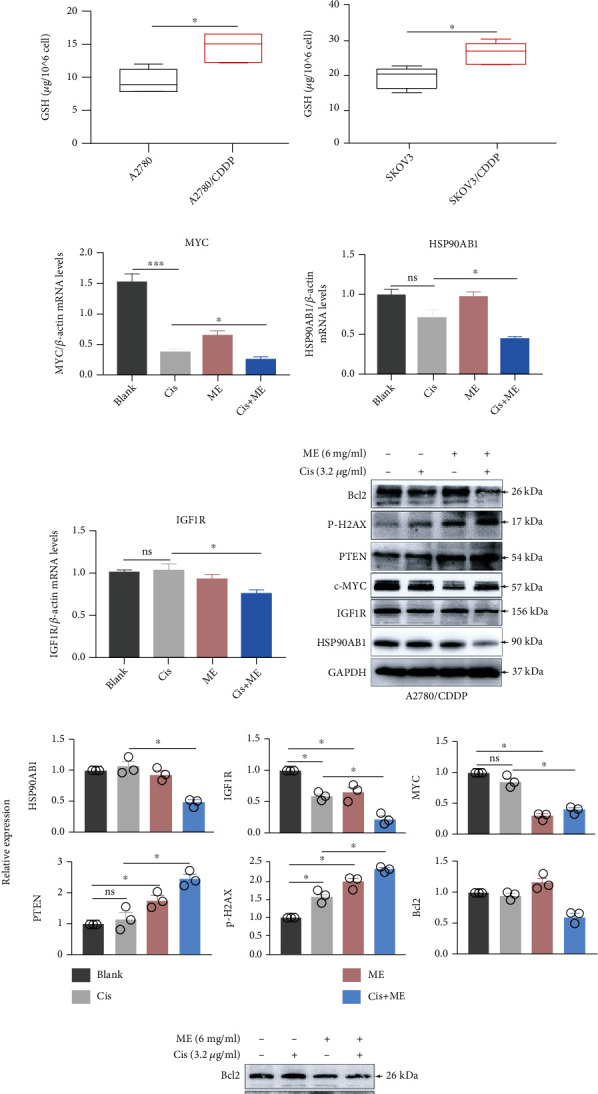
ME induces cisplatin to promote apoptosis by suppressing the expression of HSP90AB1/IGF1R in cisplatin-resistant ovarian cancer cells. (a) The expression of HSP90AB1, IGF1R, and IGFBP2 in A2780 cells and A2780/CDDP cells was assessed by western blot. (b) The lower panel shows the quantitative analysis. ^∗∗^*p* ≤ 0.01, ^∗∗∗^*p* ≤ 0.001. (c) The expression of HSP90AB1, IGF1R, and IGFBP2 in SKOV3 cells and SKOV3/CDDP cells was assessed by western blot. (d) The lower panel shows the quantitative analysis. ^∗^*p* ≤ 0.05, ^∗∗^*p* ≤ 0.01. The GSH level in A2780 cells (e) and SKOV3 cells (f) was analyzed using a reduced GSH assay kit. ^∗^*p* ≤ 0.05, ns means no significance. Relative mRNA expression of *MYC* (g), *HSP90AB1* (h), and IGF1R (i) in A2780/CDDP cells was determined by real-time PCR. ^∗^*p* ≤ 0.05, ^∗∗∗^*p* ≤ 0.001, ns means no significance. A2780/CDDP and SKOV3/CDDP cells were treated with cisplatin (3.2 *μ*g/ml) and ME (6 mg/ml) for 48 h. The protein was extracted for further analysis. (j–m) The protein expression of HSP90AB1, IGF1R, MYC, PTEN, p-H2AX, and BCL2 in A2780/CDDP cells (j) and SKOV3/CDDP cells (l) was measured by western blot. The lower panel shows the quantitative analysis of the western blot in A2780/CDDP cells (K) and SKOV3/CDDP cells (m). ^∗^*p* ≤ 0.05, ns means no significance. Drug-resistant cells were treated with cisplatin, ME, or both. The amount of GSH in A2780/CDDP cells (n) and SKOV3/CDDP cells (o) was analyzed using a reduced GSH assay kit. ^∗^*p* ≤ 0.05, ns means no significance. Three independent experiments were performed with similar results. Data are shown as the mean ± SEM.

To further explore the mechanisms by which ME enhances the sensitivity of ovarian cancer cells to cisplatin, western blot, and qRT-PCR assays were performed to examine the expression of the cancer pathway factors analyzed above. The results showed that the expression of HSP90AB1, IGF1R, and IGFBP2 in A2780/CDDP and SKOV3/CDDP cells was significantly greater than that in A2780 and SKOV3 cells (Figures 4(a)–4(d)). Consistent with this, the GSH level in cisplatin-resistant cells was also significantly increased (Figures 4(e) and 4(f)). Furthermore, we treated cells with cisplatin, ME, or both, and the expression of cancer pathway factors was assessed, including HSP90AB1, IGF1R, p-H2AX, MYC, and PTEN. The mRNA levels of *MYC*, *HSP90AB1*, and *IGF1R* were markedly decreased by ME treatment in A2780/CDDP cells (Figures 4(g)–4(i)), and ME treatment combined with cisplatin further reduced the mRNA levels of *MYC*, *HSP90AB1*, and *IGF1R* (Figures 4(g)–4(i)). Meanwhile, the protein expression of BCL2, c-MYC, IGF1R, and HSP90AB1 was dramatically decreased by ME combined with cisplatin in A2780/CDDP and SKOV3/CDDP cells. As anticipated, ME treatment significantly increased the protein expression of PTEN and p-H2AX in A2780/CDDP and SKOV3/CDDP cells (Figures 4(j)–4(m)). Furthermore, ME treatment decreased the content of GSH in A2780/CDDP and SKOV3/CDDP cells, and ME combined with cisplatin had a stronger effect (Figures 4(n)–4(o)). Together, these results demonstrate that ME treatment repressed the chemoresistance of ovarian cancer cells by attenuating the HSP90AB1/IGF1R signaling pathway and allowing cisplatin to induce DNA damage.

### 3.6. Inhibition of HSP90 ATPase Activity Enhances the Inhibitory Efficiency of ME in Chemoresistant Cells

**Figure 5:**
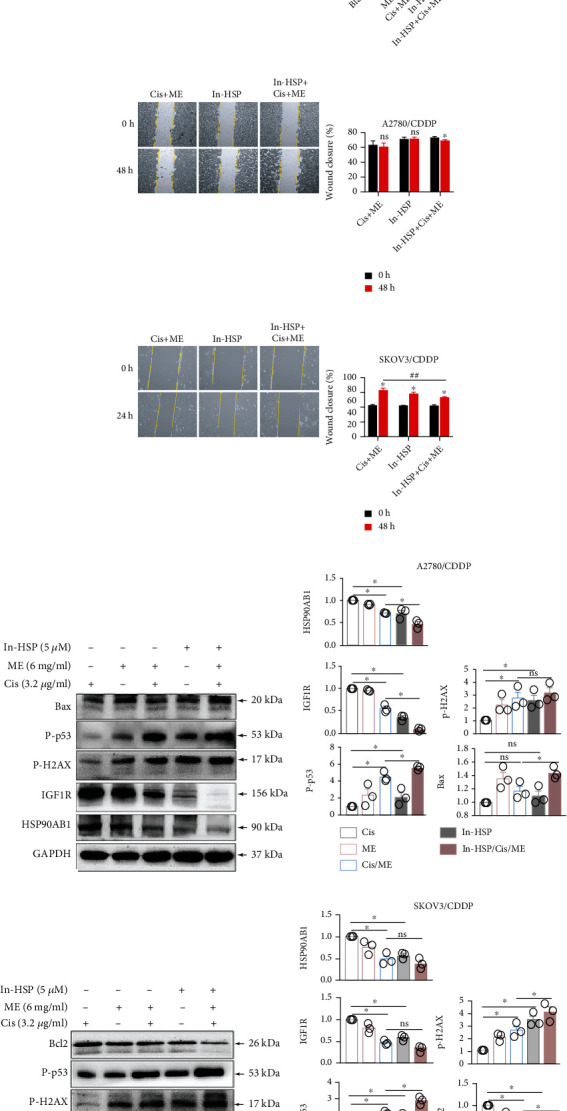
Inhibition of HSP90 ATPase activity with geldanamycin promotes apoptosis and suppresses migration in cisplatin-resistant ovarian cancer cells. A2780/CDDP cells and SKOV3/CDDP cells were pretreated with 5 *μ*M geldanamycin (In-HSP) for 1 h and then treated with cisplatin (3.2 *μ*g/ml) and ME (6 mg/ml) for 72 h. (a) A2780/CDDP cells and SKOV3/CDDP cells were double stained with Annexin V-FITC and PI and then analyzed by flow cytometry, and the quantitative analysis was located on the right. ^∗^*p* ≤ 0.05, ^∗∗^*p* ≤ 0.01, ^∗∗∗^*p* ≤ 0.001. (b, c) A2780/CDDP cells and SKOV3/CDDP cells expressing luciferase were pretreated with 150 *μ*g/ml D-luciferin for 10 min. The luciferase-positive A2780/CDDP cells (b) and SKOV3/CDDP cells (c) were analyzed using Living Image software and a GloMax® 96 Microplate Luminometer. The quantification of luciferase-positive cells is shown on the right. ^∗∗∗^*p* ≤ 0.001, ^∗∗∗∗^*p* ≤ 0.0001. Representative images of the wound healing assays of A2780/CDDP cells (d) and SKOV3/CDDP cells (e) are shown, and the quantification of the wound closure rate is on the right. ^∗^*p* ≤ 0.05 vs. 0 h; ns mean no significance vs. 0 h; ^##^*p* ≤ 0.01. (f) The expression of HSP90AB1, IGF1R, p-H2AX, p-p53, and BAX in A2780/CDDP cells was analyzed by western blot. (g) The panel on the right shows the quantitative analysis. ^∗^*p* ≤ 0.05. (h) The expression of HSP90AB1, IGF1R, p-H2AX, p-p53, and BCL2 in SKOV3/CDDP cells was measured by western blot. (i) The panel on the right shows the quantitative analysis. ^∗^*p* ≤ 0.05. (j–m) The coimmunoprecipitation assay. SKOV3/CDDP cells were treated with geldanamycin for 24 h. The expression levels of HSP90AB1 and IGF1R were measured (j), and the panel on the right shows the quantitative analysis (k). ^∗^*p* ≤ 0.05. The same cell lysates were immunoprecipitated with IGF1R, HSP90AB1, and isoform-matched immunoglobulin (IgG). (l) Western blot assays of SKOV3/CDDP cells using site-specific antibodies against HSP90AB1 and IGF1R, and the panel on the right shows the quantitative analysis (m). ^∗^*p* ≤ 0.05. Three independent experiments were performed with similar results. Data are shown as the mean ± SEM.

To investigate whether the HSP90AB1/IGF1R axis is the pathway through which ME ameliorates cisplatin resistance in ovarian cancer cells, we pretreated cells with geldanamycin to inhibit HSP90 ATPase activity prior to treatment with cisplatin and ME. We measured cell apoptosis rates using the Annexin V/PI assay, and flow cytometry analysis revealed that the apoptosis rate of A2780/CDDP cells in the geldanamycin treatment group was markedly increased compared with the cisplatin treatment group. However, geldanamycin treatment alone had no effect on the apoptosis rate of SKOV3/CDDP cells, but it significantly increased the apoptosis rates of A2780/CDDP and SKOV3/CDDP cells in the presence of both ME and cisplatin, as demonstrated by flow cytometry analysis (Figure 5(a)).

To further confirm that ME can enhance cisplatin sensitivity by targeting HSP90AB1, the cells expressing luciferase were pretreated with geldanamycin, followed by cisplatin and ME treatments, and then the cells were analyzed using Living Image software and a GloMax® 96 Microplate Luminometer. After inhibition of HSP90 ATPase activity by geldanamycin in A2780/CDDP and SKOV3/CDDP cells, the cell viability was dramatically reduced, and this was further decreased with both ME and cisplatin treatments (Figures 5(b) and 5(c)). The wound healing assay showed that geldanamycin treatment suppressed the migration of A2780/CDDP and SKOV3/CDDP cells. In addition, after inhibition of HSP90 ATPase activity, the wound closure rate following ME combined with cisplatin was significantly reduced in SKOV3/CDDP cells (Figures 5(d) and 5(e)). Moreover, western blot analysis showed that the expression of HSP90AB1 and IGF1R was significantly decreased after inhibition of HSP90 ATPase activity in A2780/CDDP and SKOV3/CDDP cells. After inhibiting HSP90 ATPase activity, treatment of cisplatin-resistant cells with both ME and cisplatin further inhibited the protein expression of HSP90AB1, IGF1R, and BCL2. Conversely, the protein expression of p-H2AX, p-p53, and BAX was remarkably increased (Figures 5(f)–5(i)). Furthermore, we performed immunoprecipitation assays and found that HSP90AB1 was physically associated with IGF1R. In addition, inhibition of HSP90 ATPase activity decreased the association of HSP90AB1 and IGF1R (Figures 5(j)–5(m)). These findings suggest that HSP90AB1 forms a complex with IGF1R that causes cisplatin resistance and that inhibition of HSP90 ATPase activity enhances the effect of ME treatment on increasing the sensitivity of chemoresistant ovarian cancer cells to cisplatin.

### 3.7. HSP90AB1 Overexpression Inhibits ME-Enhanced Sensitivity to Cisplatin in Ovarian Cancer Cells

**Figure 6:**
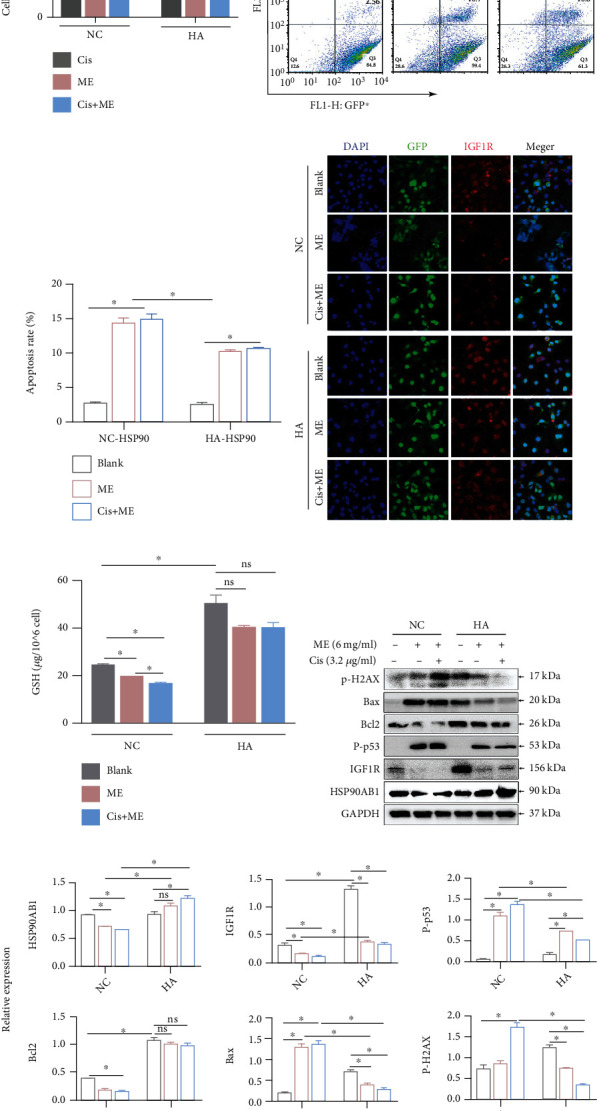
HSP90AB1 overexpression inhibits ME-induced apoptosis in SKOV3/CDDP cells. SKOV3/CDDP cells were infected for 72 h with lentiviral particles marked with GFP to overexpress HSP90AB1. The cells were then treated with cisplatin and ME. (a) Cell viability was assayed using CCK8 kits. ^∗∗∗^*p* ≤ 0.001, ^∗∗∗∗^*p* ≤ 0.0001. (b) GFP-positive SKOV3/CDDP cells were stained with PI and then analyzed by flow cytometry. (c) The lower panel shows the quantitative analysis of (b). ^∗^*p* ≤ 0.05. (d) Levels of IGF1R in GFP-positive SKOV3/CDDP cells were measured by immunofluorescence staining (600x magnification). (e) The GSH level in SKOV3/CDDP cells was analyzed using a reduced GSH assay kit. ^∗^*p* ≤ 0.05; ns means no significance. (f) The expression of HSP90AB1, IGF1R, p-p53, BCL2, BAX, and p-H2AX in SKOV3/CDDP cells was assessed by western blot. (g) The panel on the right shows the quantitative protein analysis. ^∗^*p* ≤ 0.05; ns means no significance. Three independent experiments were performed with similar results. Data are shown as the mean ± SEM.

To further evaluate the influence of HSP90AB1 on the effect of ME on enhancing the sensitivity of ovarian cancer cells to cisplatin, we overexpressed HSP90AB1 using lentivirus in SKOV3/CDDP cells. After overexpression of HSP90AB1, the cell viability was significantly greater than that of the negative control following treatment with ME combined with cisplatin (Figure 6(a)). Similarly, flow cytometry analysis of SKOV3/CDDP cells overexpressing HSP90AB1 showed a decrease in the rate of late apoptosis compared to the negative control following ME treatment (Figures 6(b) and 6(c)). As revealed by the immunofluorescence assay, ME treatment could dramatically downregulate the expression of IGF1R in negative control cells but not in HSP90AB1 overexpressing cells (Figure 6(d)). HSP90AB1 overexpression markedly increased the amount of GSH, and this was not significantly decreased by ME or by combined cisplatin and ME treatments (Figure 6(e)). Moreover, HSP90AB1 overexpression significantly reduced the inhibitory effect of ME on the expression of IGF1R and BCL2, while the opposite effects were observed for the anticancer proteins p-p53, BAX, and p-H2AX (Figures 6(f) and 6(g)). Collectively, these data demonstrate that ME treatment increases the sensitivity of ovarian cancer cells to cisplatin by suppressing HSP90AB1 expression, thereby inhibiting the proliferation, invasion, and migration of ovarian cancer cells.

## 4. Discussion

Ovarian cancer is gynecological cancer with a high mortality rate, and the majority of patients with ovarian cancer experience relapse accompanied by chemoresistance after traditional treatment [41]. Resistance to chemotherapy is a major reason for treatment failure in ovarian cancer. In this study, we found that ME is an effective adjuvant therapy that enhances the sensitivity to cisplatin of ovarian cancer cells with drug resistance. The addition of ME was shown to have a good effect in reversing chemoresistance in ovarian cancer, and this might also be the case in other cancers. Sequencing analysis revealed that HSP90AB1 and IGF1R are abnormally upregulated in drug-resistant ovarian cancer cells. As a traditional Chinese medicine, ME can prevent ovarian tumors and cause DNA damage to induce ovarian cancer cell apoptosis by inhibiting HSP90AB1/IGF1R. Inhibition of HSP90 ATPase activity with geldanamycin further enhanced the suppressive effects of ME on cisplatin-resistant ovarian cancer cells. In turn, HSP90AB1 overexpression by lentivirus effectively inhibited the enhancement of cisplatin sensitivity of ovarian cancer cells by ME. Thus, inhibition of cisplatin-induced DNA damage by HSP90AB1 overexpression is an important cause of resistance to chemotherapy in ovarian cancer, and ME can reverse cisplatin resistance by inhibiting HSP90AB1.

Most chemotherapeutic agents act by damaging the genomic DNA of tumor cells. However, drug-resistant cancer cells can overcome such DNA damage by activating alternative repair mechanisms [42]. RNA sequencing combined with bioinformatics analysis showed that *HSP90AB1* and *IGF1R*, two known drug-resistance genes, are overexpressed in A2780/CDDP cells (Figure 3), and we found that HSP90AB1 promotes IGF1R expression in ovarian cancer cells (Figure 6(f)). The overexpression of HSP90 family members has been associated with disease progression in many human malignancies, and as a member of the HSP90 family, HSP90AB1 plays multiple roles in human diseases due to the wide variety of proteins it interacts with [43, 44]. Recently, HSP90*α* and HSP90*β* have been shown to be secreted by cancer cells subjected to various stress conditions such as hypoxia, DNA damage, and growth factor stimulation [45–47]. In addition, HSP90AB1 has been reported to participate in tumorigenesis, and overexpression of HSP90AB1 has been shown to promote the angiogenesis, metastasis, and differentiation of cancer cells [48, 49]. Therefore, decreasing HSP90AB1 expression in drug-resistant ovarian cancer cells may play an effective role in promoting apoptosis in these cells.

ME is known to protect against bacterial infection and cancer [50]. Maggot therapy provides various proteolytic enzymes for the degradation of necrotic tissue in wound healing [51], and increasing evidence suggests that maggot therapy has advantages over conventional methods, especially for the treatment of wounds infected by drug-resistant bacteria [16, 17]. Our previous studies have reported that ME alleviates inflammation, oxidative stress, and fibrosis by activating Nrf2, which alleviates dextran sodium sulfate- (DSS-) induced colitis [14, 52]. However, the mechanism of ME biotherapy in cancer is still largely unknown. Our current study suggests that the suppression of HSP90AB1 by ME enhances the sensitivity of ovarian cancer cells to cisplatin, suggesting that inhibition of HSP90AB1 by ME contributes to DNA damage and apoptosis in ovarian cancer cells. Because this study is the first to explore the effect and mechanism of ME in enhancing the sensitivity of ovarian cancer cells to cisplatin, certain limitations may exist. Because ME is a crude extract that is not suitable for intravenous or intradermal injection, and for ease of quantification, we delivered MEs intragastrically to mice. The ME used in this study was a soluble mixture from the larvae of maggots, which is a limitation of this study. Future studies with purified ME components should provide us with the precise bioactive elements that confer the therapeutic effect against ovarian cancer cisplatin resistance.

Previous studies have reported that the potential mechanisms of drug resistance are related to the inhibition of apoptosis, the dysregulation of cancer stem cells, epigenetic modifications, and the induction of autophagy, hypoxia, and DNA damage and repair [4, 53]. Many cancer cells, such as ovarian cancer cells, are resistant to apoptosis and DNA damage, resulting in chemoresistance [54]. p-H2AX is a marker protein for DNA double-strand damage [55], and we evaluated the expression of p-H2AX to investigate whether DNA damage inhibition induced by HSP90AB1 overexpression is involved in cisplatin resistance. The DNA damage marker protein p-H2AX exhibited further elevated expression in HSP90AB1-inhibited cells in the presence of cisplatin and ME. Recent studies have shown that chemotherapy can activate DNA damage, and the activation of DNA damage and repair may help cells resist drug toxicity and develop resistance [56, 57]. We found that HSP90AB1 inhibition increased cisplatin-induced DNA damage. In turn, HSP90AB1 overexpression increased the amount of GSH in SKOV3/CDDP cells, which caused the inactivation of cisplatin. These results suggest that the decreased expression of HSP90AB1 increases cisplatin-induced DNA damage and thus enhances the sensitivity of ovarian cancer cells to cisplatin toxicity.

## 5. Conclusions

Our data suggest that HSP90AB1 overexpression plays an essential role in the process of cisplatin resistance in ovarian cancer. In the presence of cisplatin, ME inhibits the HSP90AB1–IGF1R interaction, thereby promoting DNA damage and apoptosis and enhancing the sensitivity of ovarian cancer cells to cisplatin. Our findings thus suggest a novel mechanism of action for ME. HSP90AB1 is a new molecular marker for chemotherapy resistance and might serve as a new drug target for ovarian cancer chemotherapy.

## Figures and Tables

**Table 1:** Primers of the human genes used in the study.

Genes	Forward	Reverse
*β*-Actin	5′-AGCGAGCATCCCCCAAAGTT-3′	5′-GGGCACGAAGGCTCATCATT-3′
MYC	5′-CCTGGTGCTCCATGAGGAGAC-3′	5′-CAGACTCTGACCTTTTGCCAGG-3′
HSP90AB1	5′-CTCTGTCAGAGTATGTTTCTCGC-3′	5′-GTTTCCGCACTCGCTCCACAAA-3′
IGF1R	5′-CCTGCACAACTCCATCTTCGTG-3′	5′-CGGTGATGTTGTAGGTGTCTGC-3′
CDKN2A	5′-CTCGTGCTGATGCTACTGAGGA-3′	5′-GGTCGGCGCAGTTGGGCTCC-3′

## Data Availability

The data that support the findings of this study are available from the corresponding author upon reasonable request.

## Abbreviations

ME: Maggot extracts
CCK8: Cell counting kit-8
HSP90AB1: Heat shock protein 90 kDa alpha, class B, member 1
IGF1R: Insulin-like growth factor 1 receptor
IGFBP2: Insulin-like growth factor binding protein 2
FBS: Foetal bovine serum
FITC: Fluorescein isothiocyanate
PI: Propidium iodide
DAPI: 4′, 6-diamidino-2-phenylindole
SDS-PAGE: Sodium dodecyl sulfate polyacrylamide gel electrophoresis
p-p53: Phosphorylated p53
DEG: Differentially expressed genes
RNA-seq: RNA sequencing
GO: Gene Ontology
KEGG: Kyoto Encyclopedia of Genes and Genomes
CDKN2A: Cyclin-dependent kinase inhibitor 2a
GSH: Glutathione
PTEN: Phosphatase and tensin homolog.

## Supplementary Materials

Supplementary 1 Supplementary Figure 1: ovarian cancer cells stably express luciferase. The cells were infected with lentiviral particles to express luciferase. A2780/CDDP (A) and SKOV3/CDDP (B) cells were treated with different concentrations of puromycin to verify the transfection efficiency of luciferase. The viability of cells was assayed using CCK8 kits.

Supplementary 2 Supplementary Figure 2: ME combined with cisplatin treatments promote apoptosis in A2780/CDDP cells. A2780/CDDP cells were treated with cisplatin (3.2 *μ*g/ml) and ME (6 mg/ml) for 48 h. A2780/CDDP cells were double stained with Annexin V-FITC and PI. The cells were imaged for apoptosis detection using an FV3000 Olympus microscope.

Supplementary 3 Supplementary Figure 3: ME combined with cisplatin treatments effectively inhibit ovarian tumor growth and metastasis to the spleen. (A) Nude mice were injected subcutaneously with SKOV3/CDDP cells to inoculate subcutaneous tumors. Photographs of the mice from each treatment group were shown. (B) Spleens from each group of intraperitoneal tumor-bearing mice were photographed to observe the tumor size in the spleen.

Supplementary 4 Supplementary Figure 4: the mRNA levels of IGF1R and CDKN2A in A2780/CDDP cells are higher than in A2780 cells. The mRNA levels of IGF1R (A) and CDKN2A (B) in A2780 and A2780/CDDP cells were analyzed by qRT-PCR assay. Three independent experiments were performed with similar results. Data are shown as mean ± SEM. ^∗∗^*p* ≤ 0.01.
