# Supplementary material for: HSP90AB1 as the Druggable Target of Maggot Extract Reverses Cisplatin Resistance in Ovarian Cancer
Source: Oxid Med Cell Longev. 2023 May 2;2023:9335440. doi: 10.1155/2023/9335440 (PMC10169247; doi:10.1155/2023/9335440)
Supplement: Supplementary 1 — Supplementary Figure 1: ovarian cancer cells stably express luciferase. The cells were infected with lentiviral particles to express luciferase. A2780/CDDP (A) and SKOV3/CDDP (B) cells were treated with different concentrations of puromycin to verify the transfection efficiency of luciferase. The viability of cells was assayed using CCK8 kits. [file 9335440.f1.docx]

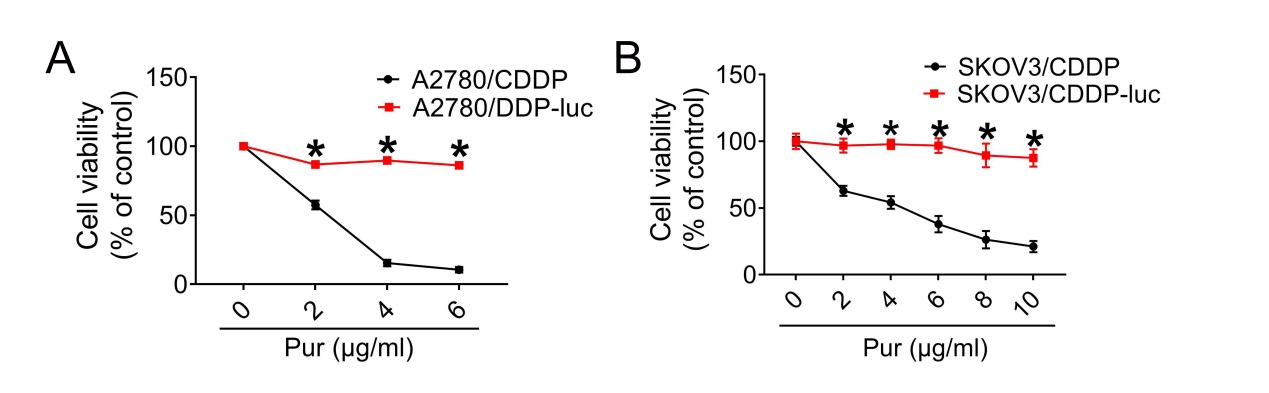


**Supplementary figure1. Ovarian cancer cells stably express luciferase.** The cells were infected with lentiviral particles to express luciferase. A2780/CDDP (A) and SKOV3/CDDP (B) cells were treated with different concentrations of puromycin to verify the transfection efficiency of luciferase. The viability of cells was assayed using CCK8 kits.
