# Supplementary material for: HSP90AB1 as the Druggable Target of Maggot Extract Reverses Cisplatin Resistance in Ovarian Cancer
Source: Oxid Med Cell Longev. 2023 May 2;2023:9335440. doi: 10.1155/2023/9335440 (PMC10169247; doi:10.1155/2023/9335440)
Supplement: Supplementary 2 — Supplementary Figure 2: ME combined with cisplatin treatments promote apoptosis in A2780/CDDP cells. A2780/CDDP cells were treated with cisplatin (3.2 μg/ml) and ME (6 mg/ml) for 48 h. A2780/CDDP cells were double stained with Annexin V-FITC and PI. The cells were imaged for apoptosis detection using an FV3000 Olympus microscope. [file 9335440.f2.docx]

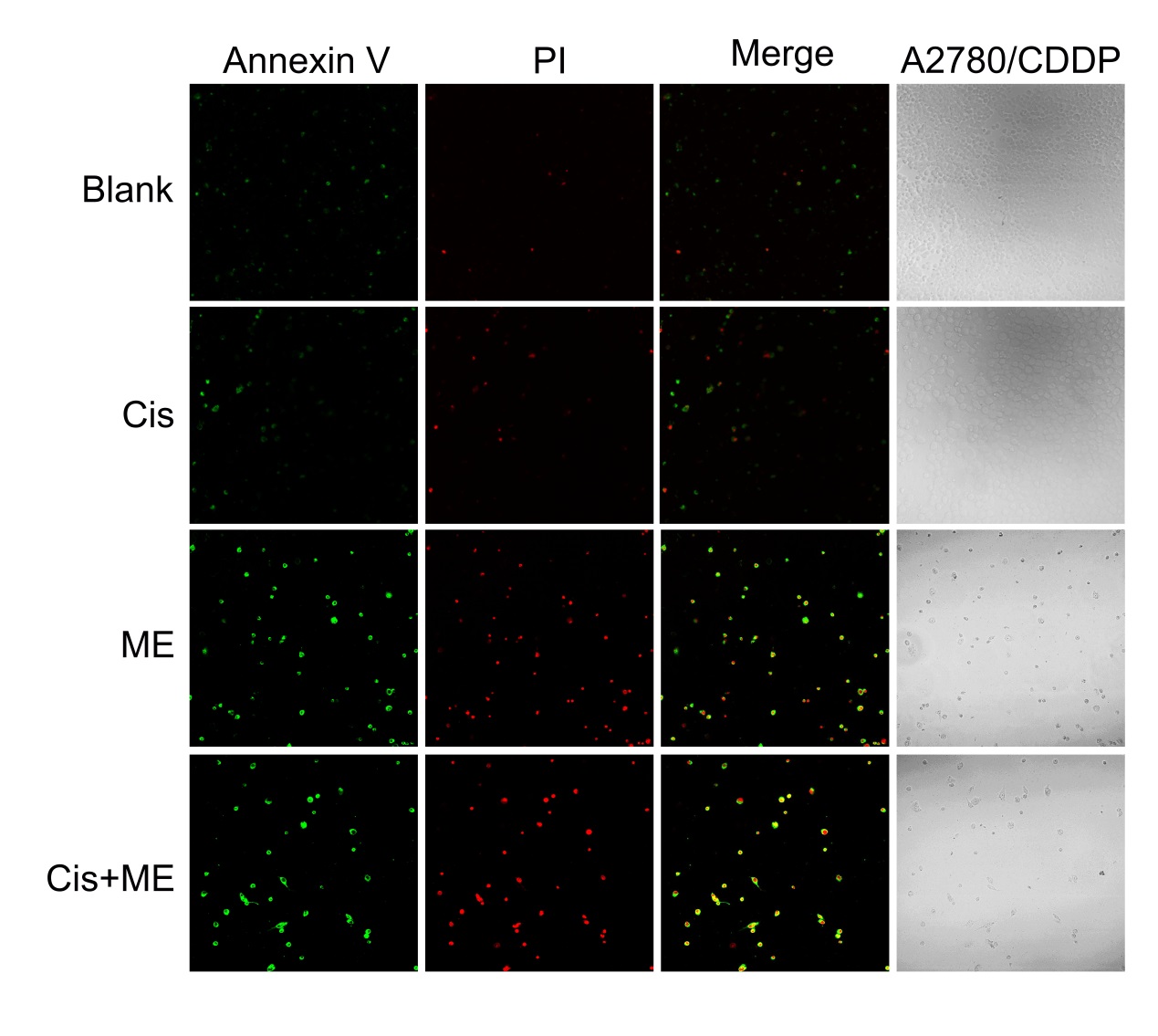


**Supplementary figure2.** **ME combined with cisplatin treatments promote apoptosis in A2780/CDDP cells.** A2780/CDDP cells were treated with cisplatin (3.2 μg/ml) and ME (6 mg/ml) for 48 h. A2780/CDDP cells were double stained with Annexin V-FITC and PI. The cells were imaged for apoptosis detection using a FV3000 Olympus microscope.
