# Supplementary material for: HSP90AB1 as the Druggable Target of Maggot Extract Reverses Cisplatin Resistance in Ovarian Cancer
Source: Oxid Med Cell Longev. 2023 May 2;2023:9335440. doi: 10.1155/2023/9335440 (PMC10169247; doi:10.1155/2023/9335440)
Supplement: Supplementary 3 — Supplementary Figure 3: ME combined with cisplatin treatments effectively inhibit ovarian tumor growth and metastasis to the spleen. (A) Nude mice were injected subcutaneously with SKOV3/CDDP cells to inoculate subcutaneous tumors. Photographs of the mice from each treatment group were shown. (B) Spleens from each group of intraperitoneal tumor-bearing mice were photographed to observe the tumor size in the spleen. [file 9335440.f3.docx]

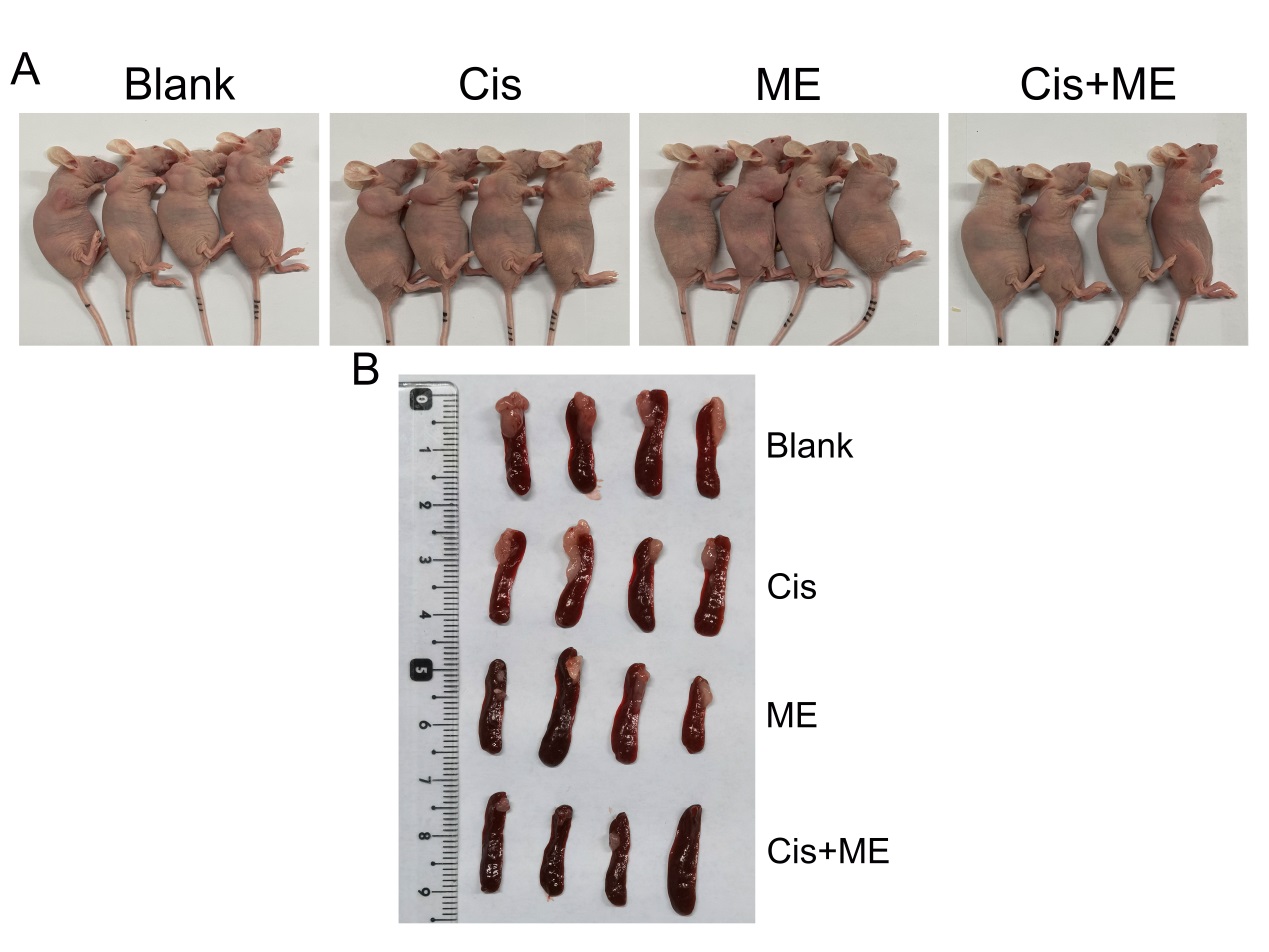


**Supplementary figure3. ME combined with cisplatin treatments effectively inhibit ovarian tumor growth and metastasis to the spleen.** (A) Nude mice were injected subcutaneously with SKOV3/CDDP cells to inoculate subcutaneous tumors. Photographs of the mice from each treatment group were shown. (B) Spleens from each group of intraperitoneal tumor-bearing mice were photographed to observe the tumor size in spleen.
