# Supplementary material for: HSP90AB1 as the Druggable Target of Maggot Extract Reverses Cisplatin Resistance in Ovarian Cancer
Source: Oxid Med Cell Longev. 2023 May 2;2023:9335440. doi: 10.1155/2023/9335440 (PMC10169247; doi:10.1155/2023/9335440)
Supplement: Supplementary 4 — Supplementary Figure 4: the mRNA levels of IGF1R and CDKN2A in A2780/CDDP cells are higher than in A2780 cells. The mRNA levels of IGF1R (A) and CDKN2A (B) in A2780 and A2780/CDDP cells were analyzed by qRT-PCR assay. Three independent experiments were performed with similar results. Data are shown as mean ± SEM. ∗∗p ≤ 0.01. [file 9335440.f4.docx]

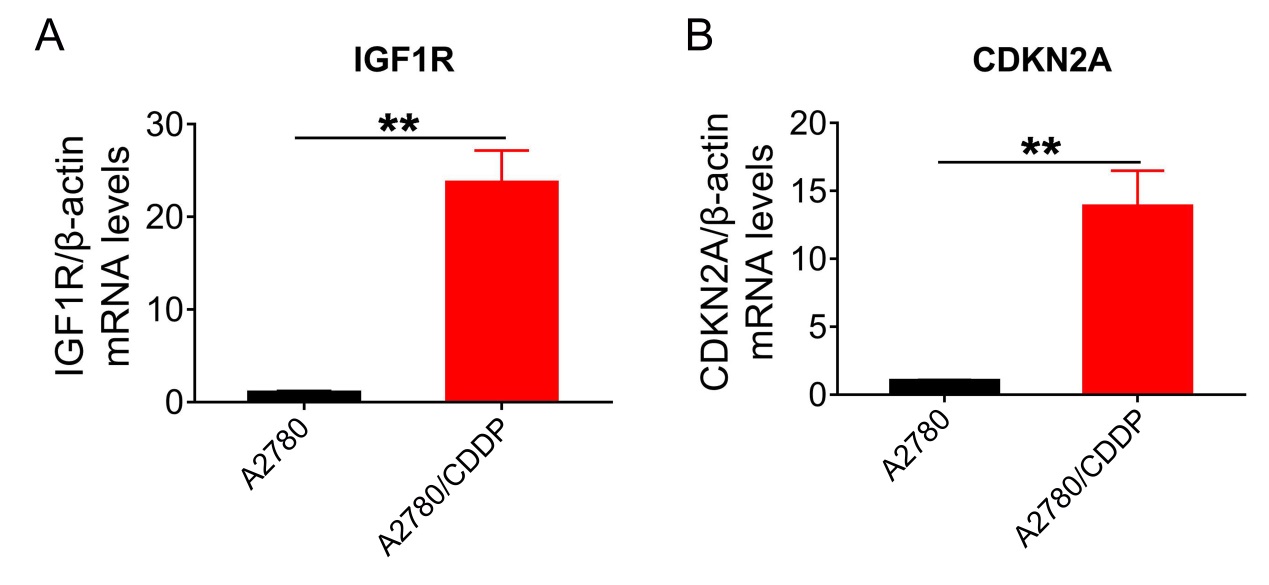


**Supplementary figure4.** **The mRNA levels of *IGF1R* and *CDKN2A* in A2780/CDDP cells are higher than A2780 cells.** The mRNA levels of *IGF1R* (A) and *CDKN2A* (B) in A2780 and A2780/CDDP cells were analyzed by qRT-PCR assay. Three independent experiments were performed with similar results. Data are shown as mean ± SEM. **p ≤ 0.01.
